# Prevalence of and Factors Associated with Driving a Vehicle with Timed-Out Inspection Certificate in Spain

**DOI:** 10.3390/ijerph19010098

**Published:** 2021-12-23

**Authors:** Luis Miguel Martín-delosReyes, Virginia Martínez-Ruiz, Mario Rivera-Izquierdo, José Pulido-Manzanero, Eladio Jiménez-Mejías, Pablo Lardelli-Claret

**Affiliations:** 1 Department of Preventive Medicine and Public Health, School of Medicine, University of Granada, Avenida de la Investigación 11, Edificio A, 8ª Planta, 18016 Granada, Spain; luismiguelmr@ugr.es (L.M.M.-d.); virmruiz@ugr.es (V.M.-R.); eladiojimenez@ugr.es (E.J.-M.); lardelli@ugr.es (P.L.-C.); 2 Centros de Investigación Biomédica en Red de Epidemiología y Salud Pública (CIBERESP), 28029 Madrid, Spain; josepuli@ucm.es; 3 Instituto de Investigación Biosanitaria de Granada (ibs.GRANADA), 18012 Granada, Spain; 4 Department of Public Health & Maternal and Child Health, Complutense University of Madrid, 28040 Madrid, Spain

**Keywords:** periodic vehicle inspection, timed-out certificate, cross-sectional, risk factors

## Abstract

This study aimed to estimate the prevalence of vehicles on the road with a timed-out vehicle inspection certificate (TOVIC) and the associations of driver, vehicle, and environmental factors with this infraction. A quasi-induced exposure approach was used in this cross-sectional study to analyze a case series comprising 51,305 drivers passively involved in clean collisions (only one infractor driver involved) between two or more vehicles registered in the Spanish National Register of Road Crashes with Victims from 2014 to 2017. The prevalence of TOVIC was estimated in the whole sample and in subgroups defined by the variables considered. Multivariate logistic regression modeling was used to obtain adjusted odds ratios for the association between TOVIC and each category of the variables. The prevalence of TOVIC was low, although significant differences were found for certain subcategories of drivers, vehicles, and environmental factors. Significant positive adjusted associations were found between TOVIC and license-related infractions, vans (compared to cars), vehicle age, and vehicle defects. Several vehicle-related factors potentially associated with a high risk of involvement in a crash were clearly related with TOVIC, which suggests the need for measures to control this non-negligible number of high-risk vehicles on the road.

## 1. Introduction

Vehicle-related factors are one of the three main groups of determinants (along with driver- and environment-related ones) classically considered in epidemiological studies of injuries caused by road crashes [1]. The contribution attributable to vehicle-related factors to the total number of road crashes has been estimated between 3% and 19% in developed countries [2,3] and 27% in developing countries [4]. This relationship justifies the implementation of periodic vehicle inspection programs aimed at detecting technical defects. Therefore, they have been imposed in different countries and regions [3,4,5,6,7]. For example, in Spain, periodic vehicle inspection, known as the Inspección Técnica de Vehículos (ITV), has been mandatory since 1985 [8]. These inspection programs are assumed to improve the environment by preventing pollutant-emitting vehicles from using the roads, and also to increase road safety. Regarding this second outcome, periodic inspections make it possibly to promptly detect (and correct) a number of vehicle defects that are known to be related with a higher risk of crashes or a higher risk of crash-related injuries (e.g., defects affecting tires, steering, brakes, lights, safety measures, etc.) [3,5,6]. The current effect of vehicle inspections on the reduction in the numbers of road crashes relies on two factors.

First, the strength of the causal association between vehicle inspection and the risk of causing a road crash is theoretically mediated by the effectiveness of inspections in reducing vehicle defects. There is a well-known relationship between the lack of compulsory inspection and the presence of defects in vehicles [6,9,10]. Moreover, some studies have observed an association between vehicle defects and the risk of road crashes [2,11,12]. However, and somewhat surprisingly, the results of previous studies designed to determine the impact of periodic inspection programs on the risk of road crashes or crash-related injuries did not show confirmative results. The first systematic review conducted on this topic in 2000 [3] concluded that most studies published before this review were too old to address the impact of periodic inspections on current vehicles, whose characteristics had changed considerably in the meantime, as evidenced by the increased warranty periods offered by car companies. In 2014, the review conducted by Jarosinski [5] suggested the existence of an underestimation of the effects of vehicle technical defects in the causal chain of road traffic injuries, because most previous studies were based on police registers, which may be affected by several sources of bias. The most recent review (2021) [7] also revealed several drawbacks in most studies [13,14,15], along with a large heterogeneity in their designs and results, which prevented drawing a definite conclusion regarding a causal relationship between periodic inspections and road safety. 

Second, the prevalence of vehicles on the road which have not passed compulsory vehicle inspection or with a timed-out vehicle inspection certificate (TOVIC) is also an important consideration in efforts to reduce road crashes. This issue has not been well addressed in previous studies [3,16], because of difficulties in collecting information about the presence or validity of inspection certificates from representative samples of vehicles on the road. 

To date, there is a lack of valid data regarding the proportion of TOVIC vehicles on roadways in Spain. However, vehicle inspection status is noted for all vehicles involved in road crashes, and TOVIC is among the infractions recorded systematically by the traffic police at crash scenes. Thus, the objectives of the present study were, first, to estimate the proportion of TOVIC vehicles on Spanish roads and, second, to identify driver, vehicle, and environmental factors associated with this offence. 

## 2. Materials and Methods

### 2.1. Study Design and Population 

We designed a cross-sectional study based on a retrospective case series initially comprising all 337,625 drivers of cars, vans and all-terrain vehicles involved in road crashes that did not involve pedestrians. The data for all cases were from the Spanish National Register of Road Crashes with Victims and were obtained for the years 2014–2017 with the ARENA2 application. This app was designed by the Spanish Traffic Directorate to provide a record of all information recorded by local, regional, and national police officers at the scene of the crash regarding the characteristics of the crash, the vehicles and the people (drivers, passengers and pedestrians) involved [17]. It has been operative since 2014 and includes all road crashes with victims occurring in Spain, except for those that occurred between 2014 and 2015 in the autonomous regions of Catalonia and the Basque Country (which were excluded for the study).

From this source population, we selected those 51,305 drivers of cars, vans, and all-terrain vehicles that were passively involved in so-called clean collisions between two or more moving vehicles (regardless of the types of other vehicles involved), defined as collisions in which only one of the involved drivers committed a driving error or a driving infraction, whereas the other (passively involved) drivers did not. According to the assumptions implicit in the quasi-induced exposure [18,19], drivers (and their vehicles) passively involved in multiple-vehicle collisions can be considered a representative sample of all drivers on the road. The distribution of all driver- and vehicle-related variables in this subgroup of road users should thus resemble that for the whole dynamic population of drivers on the road and therefore at risk of causing or being involved in a road crash. 

### 2.2. Study Variables 

We analyzed the following three groups of variables, all obtained from the National Register of Road Crashes (see the categories of each variable in Table 1 and Table 2):

- Driver-related variables: driver’s age group, sex, nationality, alcohol use, drug use, driving license status, seatbelt use.
- Vehicle-related variables: TOVIC, presence of vehicle defects, type of vehicle, years after registration, number of passengers.
- Environment-related variables: year, time of day, zone, type of road, light conditions, weather conditions and road surface.

### 2.3. Analysis

The distribution of each variable was determined for the entire sample. The prevalence of driving with a TOVIC and its 95% confidence interval (95% CI) were estimated for the whole sample and for categories of the other variables. Logistic regression modeling was used to obtain adjusted odds ratios (aOR) to estimate the adjusted association between TOVIC and the remaining study variables. All analyses were performed with Stata software version 15(StataCorp, College Station, TX, USA) [20].

## 3. Results

The prevalence of TOVIC in the whole sample was 4.94% (95% CI: 4.76–5.13). Table 1 and Table 2 summarize the distribution of vehicles and drivers across categories of the remaining variables, as well as the prevalence of TOVIC in each category. The prevalence of TOVIC was extremely high among drivers who drove without license or with a non-valid license. It was also slightly higher in the younger (from 18 to 35 years old) and older (more than 74 years old) drivers, in male drivers and in drivers who tested positive for alcohol and drugs. It was also higher for drivers for whom no information was recorded regarding safety belt use (Table 1). Regarding vehicle- and environment-related variables (Table 2), the prevalence of TOVIC was much higher in vehicles with physical defects identified by the police. It was also higher for vans and all-terrain vehicles compared to cars. Regarding the zone and the type of road, the prevalence of TOVIC was higher in accidents that occurred on urban areas compared to open roads, higher on streets or other roads (i.e., those different from highways, conventional roads, or streets) compared to highways, motorways, or conventional roads, and higher when both weather conditions and road surface were good or normal, compared to adverse or altered, respectively. It was also lower from 6:00 to 19:00 h and in daylight compared to darkness.

Table 1 and Table 2 also show the aOR values for each category of all variables included in the logistic regression model. Regarding driver-related variables, an independent association with higher prevalence of TOVIC was observed for driving without a valid license, whilst lower prevalence was found for ages between 55 and 74 years old and female sex. Vans, older vehicles, and especially vehicles with defects were related to higher prevalence of TOVIC. Finally, the environmental circumstances significantly related to higher prevalence of TOVIC were open roads (compared to urban areas), streets and other roads (compared to highways/motorways or conventional roads), and non-altered road surfaces. 

## 4. Discussion

The prevalence of TOVIC in Spain between 2014 and 2017 was low. Assuming the validity of the quasi-induced exposure approach, it was only 4.94% among all vehicles on the road. Unfortunately, there are no earlier studies with which we can compare this figure, either in Spain or in other developed countries. As noted in the Introduction, without the quasi-induced approach, attempts to estimate the prevalence of TOVIC would require data from routine checks of random samples of vehicles on the road, a regulatory procedure not currently used in Spain.

Most unadjusted differences observed in the prevalence of TOVIC across subgroups of other variables appeared to be interrelated (e.g., the high prevalence of TOVIC in males and in older ages, as older drivers in Spain are mainly men). Therefore, the most noteworthy findings were obtained in the adjusted analysis, which allows to assess the independent relationship between TOVIC and other variables. For example, applied to driver-related variables, TOVIC was strongly associated with driving without a valid driving license. If license-related infractions are markers of other high-risk driving styles, as previous studies suggest [21,22,23], high-risk vehicles can be assumed to be associated with high-risk drivers. The same reasoning could be applied for male drivers, also related with TOVIC. A higher frequency of riskier driving patterns has been reported among male compared to female drivers [24,25,26]. Therefore, the association between male sex and TOVIC might indirectly point to riskier drivers driving riskier vehicles. The same pattern is suggested by the association between TOVIC and alcohol use, although it did not reach statistical significance.

It is noteworthy that, after adjustment for other variables, the association between elderly drivers and TOVIC disappeared, confirming the explaining role of male sex on this association in the crude (unadjusted) analysis. A similar phenomenon can explain the associations between TOVIC and the place of the crash. In the crude analysis, higher prevalence of TOVIC was observed in urban areas (for the variable zone of the crash) and in streets (for the variable type of road), but these two variables are strongly correlated. Therefore, the adjusted OR estimates yielded a different pattern, revealing an independent relationship between a higher prevalence of TOVIC in open roads, streets, and non-conventional roads (i.e., neither highways, motorways, nor two lane–conventional–roads). This pattern is not surprising, if those who drive with a TOVIC tried to elude police controls, much more frequent on these types of roads, or perhaps if they tend to drive in a lesser risky environment. This later hypothesis could also explain the inverse relationship between TOVIC, and both altered road surfaces and (although it did not reach statistical significance) adverse weather conditions. Regarding these weather conditions, it should be also considered that the prevalence of TOVIC was high when this factor was not collected.

Regarding vehicle-related factors, this analysis showed a clear pattern of association between TOVIC and three subgroups of vehicles: vans, older vehicles, and vehicles with defects. Some of these associations are consistent with those reported in previous studies. For example, the association between the lack of systematic vehicle inspection and vehicle defects has been described previously [6,9]. In addition, Spanish legislation currently requires the frequency of periodic inspections to increase as vehicles become older. It is thus unsurprising that, as reported by others [2], vehicle aging is related with a higher frequency of TOVIC. Previous studies suggested that older vehicles [12,13], vans [27,28], the presence of vehicle defects [2,29], and not passing a mandatory inspection [2,14,30,31] are factors independently related with a higher risk of causing a road crash. 

The validity of our estimates relies on two assumptions. First, we assume that the quasi-induced approach is a valid method for the present analysis. Several studies have shown that in general, this assumption holds reasonably well [18,19]. A further consideration is that we imputed drivers’ responsibility for causing a crash based on whether they did or did not commit a driving error or infraction, although this imputation may also be questioned in some cases. In a collision between two or more vehicles in which only one of the drivers committed an error or infraction, the likelihood that this driver was the one who caused collision is very high. However, this assumption may not always hold, especially if the information collected by police officers at the scene of the crash was incomplete or inaccurate.

Second, we assumed that all information recorded by the police at the crash scene was correct. Because TOVIC is an objective variable, information bias was unlikely. However, police officers may, hypothetically, tend to search for (and find) more vehicle-related anomalies, such as TOVIC or vehicle defects for infractor than for non-infractor drivers. This could lead to underestimation of the prevalence of TOVIC in the latter subgroup of drivers, whom we considered to be representative of all drivers on the road. An additional potential source of bias is the accuracy (or otherwise) of other driver-related variables, such as alcohol or drug use and safety belt use. It should be noted that information for this last variable was missing in a large proportion of police accident reports. Although the reason for this is unknown, it may be related with the legal and economic consequences (i.e., loss of insurance coverage) of driving without a safety belt. It was therefore not surprising that according to most records with unknown values for some variables, the infractor drivers were in fact not using their safety belt when the crash occurred.

Third, it is possible that current prevalence of TOVIC and the magnitude of its association with the factors analyzed in the present study has changed since the study period (2014–2017). Finally, it would be quite interesting to analyze the association between TOVIC and other driver-related factors not recorded in the Spanish Register (i.e., physical conditions, fitness, etc.).

## 5. Conclusions

The prevalence of TOVIC was not high among vehicles circulating on the Spanish roads between 2014 and 2017 (less than 5%). Although this prevalence did not substantially change across categories of most driver- and environment-related variables, extremely high values were obtained for small subgroups of drivers potentially related to a high risk of being involved or causing a road crash (i.e., drivers with a timed-out driving license). Several environment-related factors, such as driving in open roads, streets, and non-conventional roads, where police controls are less frequent, were also associated with TOVIC. Males were also more frequently associated with this infraction in the adjusted estimations. Furthermore, as revealed by the multiple logistic regression models, the fact that TOVIC is positively and independently related to vans, older vehicles, and vehicles with defects implies the existence of a non-negligible volume of high-risk vehicles circulating on Spanish roads which merits attention by policymakers. Therefore, the results obtained in our study support the need to keep these at-risk vehicles off the road. To achieve this goal, routine police checks should be increased, especially for older vans with TOVIC and vehicle defects.

## Figures and Tables

**Table 1:** Distribution of variables in the sample, prevalence of TOVIC in each category, and adjusted odds ratios for the association between TOVIC and each category of driver-related variables.

Variable	Category	N	%	Prevalence of TOVIC *p* (%) 95% CI		aOR ***	95% CI	
Age * (years)	18–24	4935	9.62	5.30	4.66, 5.92	0.91	0.76	1.08
	25–34	12,350	24.07	5.43	5.03, 5.83	1.09	0.97	1.24
	35–44	14,580	28.42	4.90	4.55, 5.25	1	Reference	
	45–54	10,041	19.57	4.75	4.33, 5.17	0.91	0.79	1.04
	55–64	5652	11.02	4.40	3.85, 4.92	0.80	0.68	0.95
	65–74	2686	5.24	4.09	3.35, 4.84	0.71	0.57	0.90
	>74	873	1.7	5.49	3.40, 7.01	0.87	0.63	1.21
	Unknown	188	0.37	4.26	1.85, 8.21			
Sex **	Male	32,310	62.98	5.26	5.02, 5.50	1	Reference	
	Female	18,968	36.97	4.40	4.11, 4.69	0.87	0.79	0.96
	Unknown	27	0.05	7.41	0.91, 24.29			
Nationality	Spanish	50,395	98.23	4.95	4.76, 5.13	1	Reference	
	Foreign	888	1.73	4.95	3.53, 6.38	0.99	0.71	1.39
	Unknown	22	0.04	0				
Driving license **	Yes	50,241	97.93	4.81	4.62, 4.99	1	Reference	
	No, or not valid	1064	2.07	11.47	9.55, 13.38	2.56	2.04	3.21
Alcohol use **	No test performed	25,055	48.84	5.30	5.02, 5.57	1	Reference	
	Negative test	25,719	50.13	4.57	4.32, 4.83	0.98	0.88	1.09
	Positive test	326	0.64	7.06	4.27, 9.83	1.40	0.89	2.21
	Unknown	205	0.4	5.37	2.71, 9.40			
Drug use	No/Not tested/Not recorded	51,278	99.95	4.94	4.76, 5.13	1	Reference	
	Yes	27	0.05	7.14	0.90, 23.50	0.93	0.20	4.32
Safety belt use **	Yes	43,499	84.79	4.68	4.48, 4.87	1	Reference	
	No	377	0.73	4.51	2.41, 6.61	0.75	0.45	1.25
	Unknown	7429	14.48	6.52	5.96, 7.10			

* Chi-squared test: *p* < 0.05. ** Chi-squared test: *p* < 0.01. *** aOR: Adjusted odds ratio for the association between TOVIC and each category of the other variables. To obtain aOR estimates we included in the logistic regression model TOVIC plus all other study variables and excluded from the model all records with unknown values for any variable.

**Table 2:** Distribution of variables in the sample, prevalence of TOVIC in each category, and adjusted odds ratios for the association between TOVIC and each category of vehicle- and environmental-related variables.

Variable	Category	N	%	Prevalence of TOVIC *p* (%) 95% CI		aOR ***	95% IC	
Type of Vehicle **	Car	45,474	88.63	4.65	4.46, 4.84	1	Reference	
	Van	4560	8.89	7.79	7.01, 8.56	1.80	1.58	2.06
	All-terrain	1271	2.48	5.27	4.04, 6.50	1.09	0.82	1.45
Vehicle defects **	None	51,088	99.58	4.86	4.68, 5.05	1	Reference	
	Any	212	0.41	24.06	18.29, 29.82	5.58	3.94	7.90
	Unknown	5	0.01	20.00	0.01, 71.6			
Passengers	Driver only	29,624	57.74	5.02	4.77, 5.26	1	Reference	
	Any other	21,681	42.26	4.85	4.56, 5.13	0.96	0.87	1.05
Vehicle age	One-year increase	Mean: 10.10	SD: 5.75			1.07	1.06	1.08
	Unknown	20	0.09	0				
Year	2014	10,370	20.21	4.98	4.56, 5.39	1	Reference	
	2015	12,820	24.99	4.72	4.35, 5.09	0.93	0.81	1.07
	2016	13,699	26.7	4.95	4.59, 5.31	0.91	0.80	1.04
	2017	14,416	28.1	5.12	4.76, 5.48	0.94	0.82	1.07
Zone **	Urban	21,327	41.57	5.48	5.18, 5.79	1	Reference	
	Open road	29,978	58.43	4.56	4.33, 4.80	1.90	1.33	2.72
Type of Road **	Highway/Motorway	11,390	22.2	4.42	4.05, 4.80	1	Reference	
	Conventional road	18,160	35.4	4.46	4.16, 4.76	0.94	0.84	1.06
	Street	19,588	38.18	5.63	5.31, 5.95	2.02	1.39	2.93
	Other roads	2167	4.22	5.54	4.57, 6.50	1.44	1.11	1.85
Time of day * (24-h clock)	0–5	1573	3.07	6.17	4.98, 7.36	0.94	0.84	1.06
	6–11	13,070	25.48	4.79	4.42, 5.16	1	0.89	1.13
	12–19	21,369	41.65	4.76	4.48, 5.05	1	Reference	
	20–24	15,293	29.81	5.20	4.85, 5.56	1.03	0.90	1.18
Light conditions	Daylight	38,570	75.18	4.78	4.57, 4.99	1	Reference	
	Dawn/Dusk without artificial lighting	1663	3.24	4.93	3.89, 5.97	0.97	0.75	1.26
	Dawn/Dusk with artificial lighting	1388	2.71	5.40	4.21, 6.59	1.14	0.86	1.50
	Dark with artificial lighting on	5874	11.45	5.55	4.96, 6.14	1.15	0.97	1.38
	Dark with artificial lighting off	648	1.26	5.56	3.79, 7.32	1.33	0.92	1.92
	Dark without artificial lightning	3162	6.16	5.53	4.74, 6.33	1.16	0.96	1.42
Weather Conditions **	Good	42,995	83.8	5.17	4.96, 5.38	1	Reference	
	Adverse	8102	15.79	3.74	3.33, 4.15	0.82	0.68	1
	Unknown	208	0.41	5.77				
Road surface **	Normal	45,022	87.75	5.14	4.94, 5.35	1	Reference	
	Altered	6205	12.09	3.55	3.09, 4.01	0.74	0.60	0.93
	Unknown	78	0.15	2.56	0.31, 8.96			

* Chi-squared test: *p* < 0.05. ** Chi-squared test: *p* < 0.01. *** aOR: Adjusted odds ratio for the association between TOVIC and each category of the other variables. To obtain aOR estimates we included in the logistic regression model TOVIC plus all other study variables and excluded from the model all records with unknown values for any variable.

## Data Availability

Data from this study should be requested to the Spanish Traffic Directorate (Dirección General de Tráfico).
